# Enhancement of Particle Alignment Using Silicone Oil Plasticizer and Its Effects on the Field-Dependent Properties of Magnetorheological Elastomers

**DOI:** 10.3390/ijms20174085

**Published:** 2019-08-21

**Authors:** Muntaz Hana Ahmad Khairi, Abdul Yasser Abd Fatah, Saiful Amri Mazlan, U. Ubaidillah, Nur Azmah Nordin, Nik Intan Nik Ismail, Seung Bok Choi, Siti Aishah Abdul Aziz

**Affiliations:** 1 Engineering Materials and Structures (eMast) iKohza, Malaysian-Japan International Institute of Technology, Universiti Teknologi Malaysia, Jalan Sultan Yahya Petra, Kuala Lumpur 54100, Malaysia; 2 Department of Engineering, Razak Faculty of Technology and Informatics, Universiti Teknologi Malaysia, Jalan Sultan Yahya Petra, Kuala Lumpur 54100, Malaysia; 3 Mechanical Engineering Department, Faculty of Engineering, Universitas Sebelas Maret, Jl. Ir. Sutami 36A Kentingan Jebres, Surakarta 57126, Indonesia; 4 National Center for Sustainable Transportation Technology (NCSTT), Bandung 40132, Indonesia; 5 Technology and Engineering Division, Advanced Rubber Technology Unit, Rubber Research Institute Malaysia (RRIM), Sungai Buloh 47000, Selangor Darul Ehsan, Malaysia; 6 Department of Mechanical Engineering, Inha University, 253, Yonghyun-dong, Namgu, Incheon 402-751, Korea

**Keywords:** magnetorheological elastomer, alignment, anisotropic, silicone oil

## Abstract

The existing mold concept of fabricating magnetorheological elastomer (MRE) tends to encounter several flux issues due to magnetic flux losses inside the chamber. Therefore, this paper presents a new approach for enhancing particle alignment through MRE fabrication as a means to provide better rheological properties. A closed-loop mold, which is essentially a fully guided magnetic field inside the chamber, was designed in order to strengthen the magnetic flux during the curing process with the help of silicone oil (SO) plasticizers. The oil serves the purpose of softening the matrix. Scanning electron microscopy (SEM) was used to observe the surface morphology of the fabricated MRE samples. The field-dependent dynamic properties of the MREs were measured several ways using a rheometer, namely, strain sweep, frequency sweep, and magnetic field sweep. The analysis implied that the effectiveness of the MRE was associated with the use of the SO, and the closed-loop mold helped enhance the absolute modulus up to 0.8 MPa. The relative magnetorheological (MR) effects exhibited high values up to 646%. The high modulus properties offered by the MRE with SO are believed to be potentially useful in industry applications, particularly as vibration absorbers, which require a high range of stiffness.

## 1. Introduction

Magnetorheological (MR) materials fall under the class of smart materials, due to their rheological properties that can be actively changed continuously, rapidly, and reversibly, by manipulating the strength of the magnetic field. MR materials such as MR fluid (MRF), MR elastomer (MRE), MR grease (MRG), and MR foam have been extensively investigated as potential materials for various applications, especially for the application of vibration absorbtion control devices. This is due to their unique property that depicts fast real-time responses toward different intensities of magnetic fields [1]. Among these MR materials, MRF has established itself in applications especially related to the hydraulic devices in the automotive sector, such as torque and force transmitting devices [2], dampers [3,4], clutches [5], and brakes [6]. On the other hand, for the past few years, MRE has attracted an increasing interest as it is able to exhibit a desirable performance owing to the stability of magnetizable particles in a solid-based matrix [7]. Critical issues associated with MRF, such as leaking, caking, and the sedimentaion of particles, make it less desirable than MRE, where the latter is known as a much easier material to handle [8].

MRE is a polymer composite which mainly consists of a rubber matrix, ferromagnetic particles as a reinforcement phase, and optional additives for acquiring specific properties, such as increased stability and a much more superior bonding between the matrix and the magnetic particles [9]. Depending on the curing process, it can be classified into two categories—isotropic MRE, which consists of randomly distributed particles, and anisotropic MRE, which is made up of aligned particles in the rubber matrix [10]. Due to this fact, anisotropic MRE possesses a closer gap between the magnetic particles, as compared to that in isotropic MRE. This can lead to strong interactions between the particles, which makes it much more sensitive toward the change in the magnetic field’s intensity. Hence, it can provide a much higher modulus range through alteration of the magnetic field, leading to much higher MR effects.

The ability of viscoelastic materials to store energy is known as storage modulus (G’), while the loss factor (tan δ) refers to the damping properties of a certain material which take place due to the energy dissipation in the MRE when a force is applied. Fundamentally, the shear storage modulus (G’), and the loss factor (tan δ) are the principal parameters that describe the rheological properties and subsequently assist in measuring the performance of resultant MREs. A higher G’ means that the MRE has less deficit energy, and the material is much harder. With regards to the loss factor, a material with a greater loss factor (tan δ) than the value of 1 exhibits a greater damping performance. Due to the capability of reversibly changing properties, MRE has become a promising material for a broader range of industry applications such as vibration and noise reduction in the automotive sector [11] and seismic structures [12], stretchable sensors [13], and medical devices [14].

It has been observed that natural [15] and synthetic elastomers [16] have been widely used as a matrix base for MREs. The type of matrix used is widely known to influence the MR effects of the MRE. The MR effect is smaller in the case of a hard matrix phase (stiffer). In general, the process of vulcanizing MRE at a high temperature is required for developing the natural rubber-based matrix. However, for silicone rubber-based MREs, it is satisfactory to carry out the vulcanization at room temperature. In addition, the vulcanization of silicone rubber-based MREs is less complex compared to that of natural rubber-based MREs. Therefore, silicone-based materials that have undergone room temperature vulcanizing (RTV), such as RTV silicone 106 and 704, are widely used as the MRE matrix. In fact, the post-curing shrinkage phenomenon for samples made of these types of silicones can be neglected when compared to that of natural rubber, which tends to shrink with drying or curing [17]. The samples typically exhibited higher tear strength and medium shore hardness (approximately 40), and offered better heat and aging resistance [18].

The main factors that influence the MR effects are the particle’s shape, size, and volume content, the orientation of the magnetic particles, and the stiffness of the matrix. Lokander and Stenberg [19] showed experimentally that the size and shape of the magnetic particles have an influence on the MR effect of isotropic nitrile rubber-based MREs. The MREs that used ASC300 iron particles with a size of less than 60 had an MR effect which was much larger than the MREs containing carbonyl iron particles (CIPs) in the range of 3.9–5.0 µm. Li and Zhang [20] showed that for a particular ratio of bimodal magnetic particle mixture, the MR effects of the silicone-based MREs could be enhanced. In terms of particle types, CIPs are mainly used by researchers as soft magnetic materials [21], as compared to nickel [22], cobalt [23], magnetite [24], and iron sand [25], which is much harder. This is due to its high permeability, high magnetic saturation, and low remanence, which leads to a large MR effect [26]. In another study, Lokander and Stenberg [19] demonstrated that the maximum MR effect on the MRE could be achieved when the content of the magnetic particles was at about 70 wt%.

It is known that the higher the particle loading in MRE, the higher the storage modulus, thereby enhancing the absolute and relative MR effects [27]. However, too high a particle loading significantly increases the viscosity of the polymer composite, which in return results in a higher initial storage modulus, simultaneously reducing the MR effect. Therefore, to reduce the respective initial storage modulus, the use of a certain plasticizer is introduced as an additive to the MRE. The plasticizer improves the interaction between the particles, as it helps provide less restriction from the plasticized matrix. It has been found in [28] that the initial storage modulus could be reduced and, consequently, the relative MR effects could be enhanced using silicone oil (SO) as a plasticizer in MRE. In fact, there is another approach for improving the MR effect using plasticizers besides the adjustment of the initial storage modulus [28]. The MR effect can be enhanced by optimizing the curing flux, so as to strengthen the particle alignment with the help of a plasticizer.

The main contribution of this work is to propose a new approach for enhancing particle alignment in MRE fabrication using SO plasticizers. More specifically, a closed-loop mold was used in this work to achieve closer particle alignment in anisotropic MRE, which is assumed to provide better rheological characteristics depending on the magnetic field intensity. The mold design should be sufficiently spacious to allow uniform magnetic flux inside and outside the chamber, and to ensure that the applied magnetics of the flux flows inside the chamber. The fabrication of anisotropic MRE with the addition of SO was carried out with 0-degree magnetic particle alignment, parallel to the direction of the magnetic field, and across the MRE samples. The common anisotropic 0-degree was chosen because the fabrication of MRE via a close directed alignment mold is much simpler, and the energy consumption for the 0-degree alignment to occur is lower, but still shows a high MR effect.

The SO was added to MRE by varying the weight percentages for 0, 5, 10 and 15 wt% in order to investigate the effect of different SO contents on the performance of the MREs. The isotropic MRE was fabricated as a comparative reference in terms of MR effect, and across morphological aspects. After fabricating the proposed MRE, observations via scanning electron microscopy (SEM) were made to understand the particle alignment in the samples. Subsequently, the field-dependent dynamic viscoelastic properties of the MREs were measured using a rheometer across several methods, namely, strain sweep, frequency sweep, and magnetic field sweep. The measured results were compared, and the effectiveness of the proposed MRE associated with the SO plasticizer was validated to determine the enhancement of the absolute and relative MR effects.

## 2. Results and Discussion

### 2.1. Tensile Strength of MRE with SO

**Figure 1:**
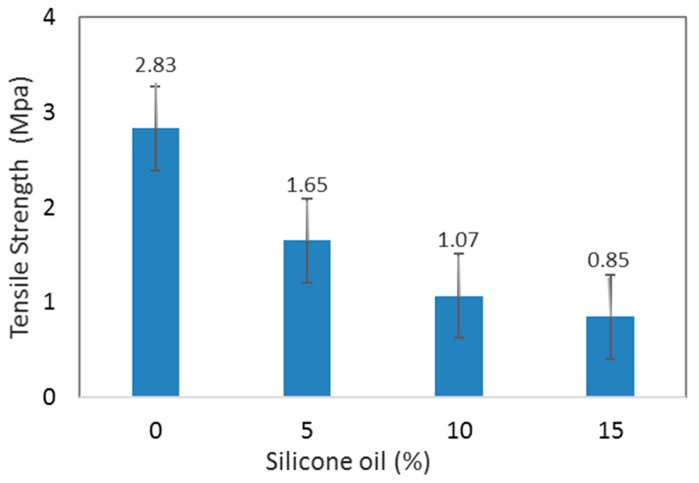
Tensile strength of isotropic MRE as a function of the silicone oil (SO) plasticizer content.

MRE showed a higher tensile strength corresponding to a stronger MRE. Tensile strength is defined as the maximum stress which the material can withstand before it ruptures or fails. In other words, how much can MRE endure before it is stretched toward breakage or failure [29]. Figure 1 shows the tensile strength of the isotropic MREs as a function of SO content. It can be observed that the tensile strength of the MRE reduced with the addition of SO, which was consistent with the general expectation for a plasticized polymer [29]. By incorporating the plasticizer, particularly SO into the polymer system, it reduced the viscosity or cross-link density of the MRE in the rubber matrix, which softened due to an increase in the rubber chain’s mobility.

This phenomenon also facilitated the movement of the rubber molecular chains to each other by giving them an internal lubricity. Since the network of the system’s cross-link density decreased, the distance between the ends of the two rubber molecules increased, and thus, the tensile strength decreased. In the current study, the experiment results revealed that the tensile strength of MRE without SO was 2.9 MPa. However, by adding SO up to 5 wt%, the tensile strength decreased to 1.65 MPa, and further decreased to 1.1 and 0.9 MPa with 10 wt% and 15 wt% SO, respectively. Although the MRE might exhibit a reduction in strength and stiffness because of the plasticizer, the polymer material is believed to be more useful in certain applications that require more flexible or softer materials [30].

### 2.2. Morphology of MRE with SO

**Figure 2:**
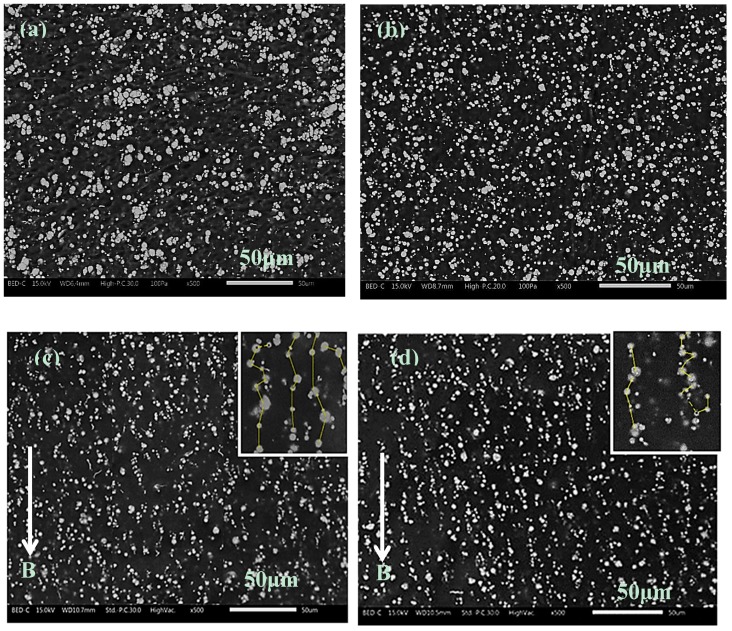
Fractured surface morphology of MREs: (**a**) isotropic MRE with 0 wt% SO and (**b**) isotropic MRE with 15 wt% SO; (**c**) anisotropic MRE with 0 wt% SO and (**d**) anisotropic MRE with 15 wt% SO.

The cross-sectioned area surface morphologies of the isotropic and anisotropic MRE are presented in Figure 2. It can be observed that for the isotropic MRE, with or without SO, the CIPs are dispersed randomly in the matrix, as shown in Figure 2a,b, respectively. However, the presence of 15 wt% of SO improved the distribution of the CIPs. No large aggregates are seen in the fractured surface of the MRE, as shown in Figure 2b. On the other hand, for the anisotropic MRE without SO under curing of the magnetic field of 300 mT as shown in Figure 2c, the CIPs are observed to have aligned in the vertical direction, forming the chain-like structure following the direction of the magnetic field. When the magnetic field is applied to the pre-polymer, the interaction and attraction among the CIPs make them align along the magnetic field. As the curing reaction takes place, the CIPs’ chain-like structure becomes fixed and locked in the matrix. Similarly, the micrograph of the anisotropic MRE with 15 wt% SO is shown in Figure 2d. The chain-like structures of the SO anisotropic MRE are much clearer than those of MRE without SO; the average distance between CIPs is 4.083 μm as compared to MRE without SO that has an average distance between CIPs of 6.687 μm as determined by using ImageJ analysis (insets in Figure 2c,d). This indicates that the introduction of SO enhanced the distribution of the isotropic MRE, at the same time helping the CIPs to align in the case of anisotropic MRE.

Based on the rheological behavior of the SO anisotropic MRE dispersions, it is concluded that the incorporation of SO can reduce the viscosity of the CIP dispersions. Thus, the resistance of the CIP orientation in the pre-polymer is reduced, resulting in a more obvious chain-like structure to provide better rheological properties [31]. Therefore, the role of SO before and during the curing process is to reduce the viscosity of the matrix, and makes the CIP alignment easier, while SO makes the MRE into a soft, flexible, and elastic material after curing. This morphological result agrees with the work undertaken by Tian and Nakano [31], in which the addition of SO to MRE improved the chain alignment, as compared to that of non-SO-based MRE samples. The correlation between particle structures and orientation to the rheological properties has been reported by Davis [32], whose study used the magnetic dipole theory to compare the rheological behavior before and after dispersion of magnetic particles. In another study, the distribution of the distance between adjacent particles was proposed by Suo et al. [33], using a magnetic dipole model based on chi-squared distribution. All mathematical models mentioned above considered the distance and magnetic dipole affecting the rheological behavior of MRE from the microstructure prospective.

### 2.3. Rheological Properties with SO

#### 2.3.1. Magnetic Field Sweep

**Figure 3:**
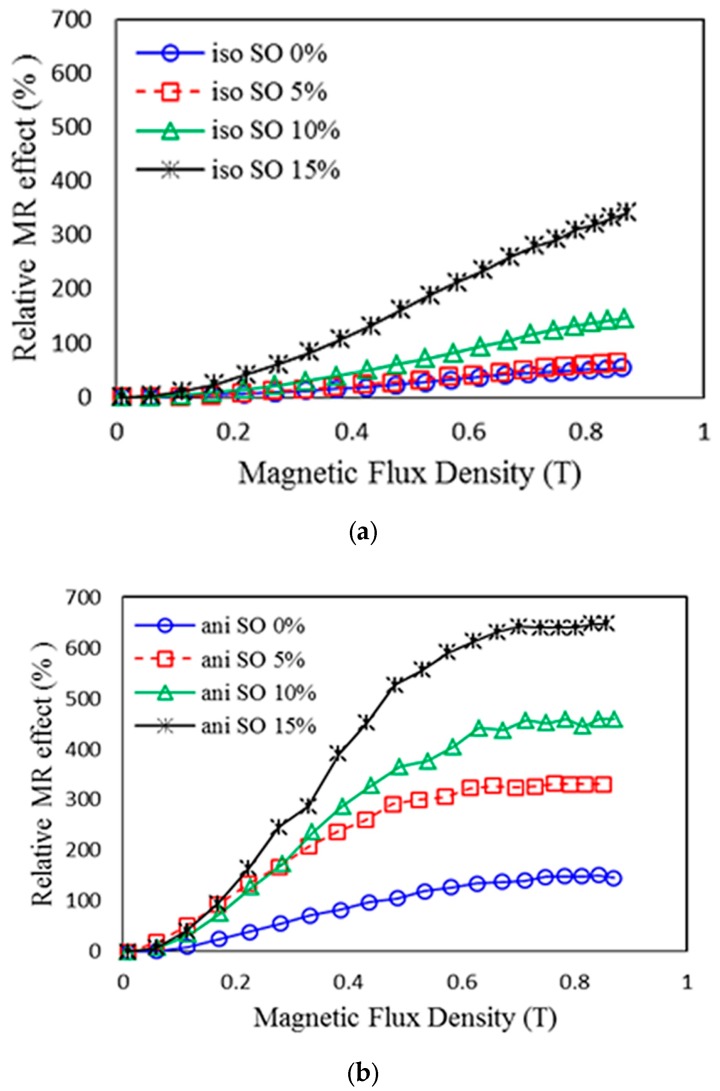
Relative MR effect for (**a**) isotropic MRE and (**b**) anisotropic MRE as a function of sweep magnetic field at various contents of SO.

The MR effect is a crucial parameter for evaluating the MRE’s performance, which is the ratio of the magneto-induced modulus and the initial modulus. The MR effect is evaluated by the following equation:

(1) $$G_{effect}=\frac{\left(G_{max}-G_{0}\right)}{G_{0}}\;\times \;100\%$$

where *G*_0_ is the zero-field modulus and *G_max_* is the maximum modulus when the MRE is imposed at magnetic saturation. Meanwhile, SO is an effective plasticizer for silicone rubber, which can reduce the molecular interaction and soften the matrix. The effects of SO content on the absolute and relative MR effect under different magnetic fields of isotropic and anisotropic MRE-based silicone rubber with 70 wt% CIPs are shown in Figure 3.

The related zero-field modulus and MR effect data are summarized in Table 1. For both isotropic and anisotropic MRE, the zero-field modulus decreases significantly with increasing SO content. For isotropic MRE, the absolute MR effect shows a slight increase with increasing SO content, while the relative MR effect increases from 55% to 343%, which is attributed to the significant decrease in the zero-field modulus with the addition of the plasticizer. For anisotropic MREs, both the absolute and relative MR effects increase greatly with SO content. Moreover, the magneto-induction for the anisotropic MREs reaches the saturation state as shown in Figure 3b. It can be seen from the experimental results that the absolute and relative MR effects of the anisotropic MRE with 15 wt% (the weight ratio of CIP to the silicone rubber to the SO is 70:15:15) reach 0.8 MPa and 646%, respectively, which are 1 and 4 times that of MREs without plasticizers. This exceeds the results from Tian et al. [31] which depicted a value of 0.3 MPa for the absolute MR effect [31].

The associated improvement of the relative and absolute MR effects was achieved by using the closed-loop mold concept, which enhanced the CIP alignment by providing more magnetic flux into the MRE. Furthermore, the CIPs’ alignment and movement were much easier and in close quarters to one another during the curing process, when the content of plasticizer was much higher at 15wt%. Therefore, the lower zero-field modulus and improved interaction between the aligned CIPs (using the closed-loop mold concept) with the addition of SO are two main factors for the improvement of the MR effect in the anisotropic type of MRE.

**Figure 4:**
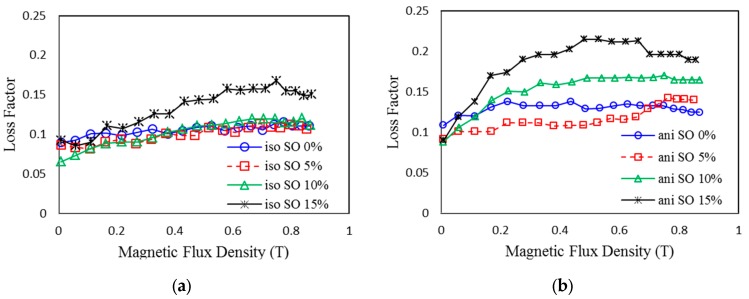
Loss factor of MRE with different contents of SO under different magnetic fields: (**a**) isotropic MRE and (**b**) anisotropic MRE.

Figure 4 shows the loss factor of the isotropic and anisotropic MRE samples under different magnetic fields. As it can be seen, increasing the SO also increased the loss factor. It was slightly higher for anisotropic samples. Chen et al. [34] demonstrated that the loss factor or damping of the MRE originates mainly from the interfacial friction between the magnetic particles and the rubber matrix. Increasing the SO lowered the MRE viscosity; this led to the enhancement of the sliding of the molecular chains toward the matrix. The input energy from the stress was dissipated from the interfacial friction, which caused an increment in the damping properties of the MRE [35]. It can also be seen from Figure 4 that the loss factors of the samples show a tendency to increase, and then decrease, as the magnetic flux density is increased. For the higher SO content of 15 wt%, this tendency seems to be much clearer. When the magnetic field was low, the force generated between the particles increased the energy dissipation, which was caused by the interfacial sliding, playing a key role in the increment of the energy loss [34]. Furthermore, the interaction of matrix and particles is also relatively weak at a low magnetic field; thus, the sliding energy dissipation is produced easily under shear stress.

**Figure 5:**
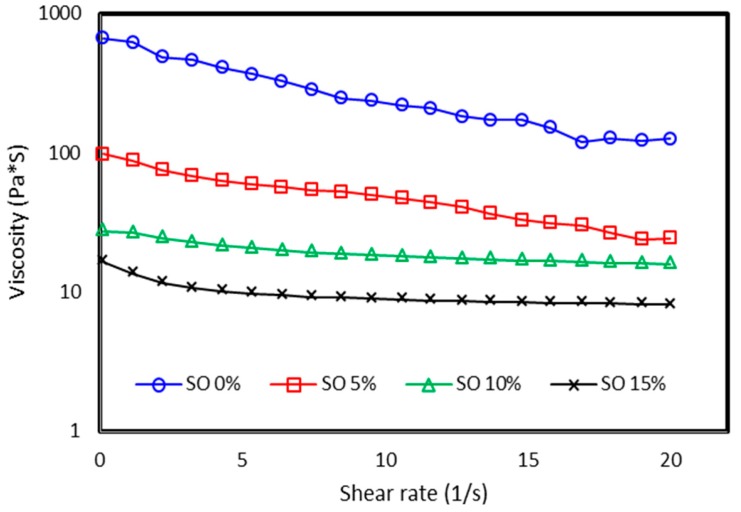
Viscosity of the uncured MREs with different contents of SO under different shear rates.

Moreover, the sliding energy dissipation is easier for soft matrix (MRE with SO) due to the high mobility between the molecular chains in the low cross-link density, as compared to non-SO samples. Therefore, the loss factor has an increasing tendency in low magnetic fields, particularly for MREs with a higher content of SO. However, when a high magnetic field is applied to the MRE sample, the strong interaction force between the particles and the rubber matrix can decrease the sliding displacement. Thus, the energy dissipation is reduced, and the loss factor is decreased when the magnetic field is increased further. This is because at higher magnetic fields, the rubber is more constrained due to the interaction between the magnetic particles. Therefore, the energy dissipation of the molecular rubber is decreased, which reduces the loss factor further. The tendency for the decline in the MRE loss factor agrees well with previous researchers [36]. Meanwhile, the viscosity of the matrix determines the elasticity of the MRE. The dispersion and homogeneous distribution of the CIPs in the rubber matrix are the most significant steps in the fabrication of the MRE. The viscosity of the matrix has a great effect on the dispersion result of the magnetic particles [36]. Figure 5 shows the viscosity of the uncured MREs with different contents of SO under different shear rates. For rubber without the SO, the CIPs are hard to disperse due to the higher viscosity of the rubber matrix.

In contrast, with the addition of the SO, particularly up to 15 wt%, the CIPs are found to disperse well in the rubber matrix, as seen from the SEM image in Figure 2b. Furthermore, the viscosity of the matrix decreases as the SO content is increased. Therefore, it is believed that for the case of plasticized anisotropic MRE, the lower viscosity of the rubber matrix helps the CIPs to align easily following the applied magnetic field, which results in superior damping properties. This analysis agrees well with the SEM results. The CIPs in samples with SO form an aligned structure in the anisotropic MRE (Figure 2d), and the CIPs are seen to have been dispersed homogeneously and are well embedded in the rubber matrix by having less agglomeration structures for the isotropic MRE (Figure 2b).

#### 2.3.2. Strain Sweep

**Figure 6:**
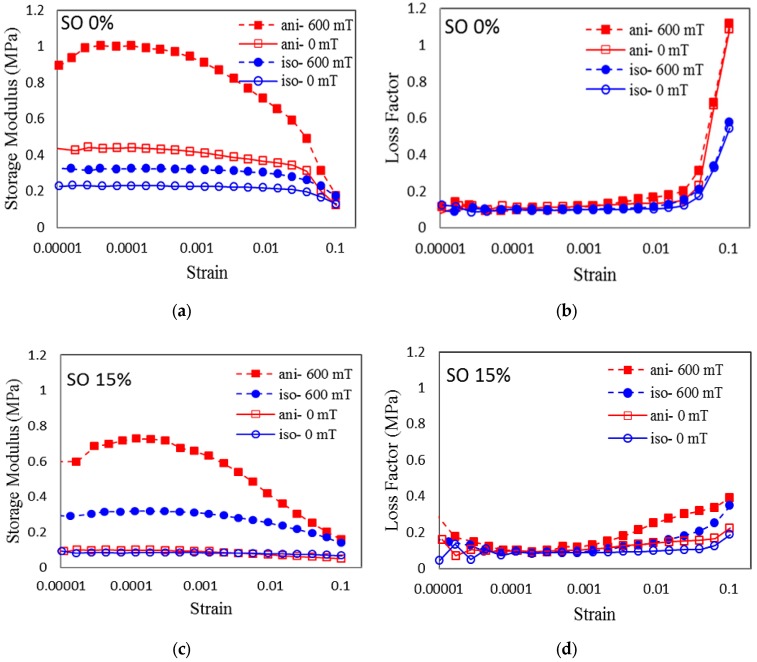
Storage modulus and loss factor of MRE with (**a**,**b**) 0 wt% of SO and (**c**,**d**) 15 wt% of SO as a function of oscillation strain at various magnetic fields.

The strain amplitude sweep test was carried out to investigate the effect of shear strain on the dynamic viscoelastic modulus of the MRE samples. This mechanism applied a fixed oscillatory strain to the specimen and measured the amplitude and phase of the output force, from which the shear storage modulus was calculated. The linear viscoelastic (LVE) region plays a role in predicting the behavior of MRE samples within an absolute limit. Figure 6 shows the storage modulus of the MRE samples with the LVE region as a function of the strain amplitude.

Selected plots of the rheological properties are presented as a function of the oscillation strain across different magnetic fields. Figure 6a–d demonstrates the effects of strain at 0 and 600 mT of the applied magnetic field, to the isotropic and anisotropic SO-based MREs for the lowest and highest SO content, 0 and 15 wt%, respectively. As shown in Figure 6a,b, all samples exhibited strain-dependent behavior, in which the shear storage modulus decreased with increasing strain. A slight decrease in the shear storage modulus with increasing strain was observed for the isotropic MRE samples with 0 wt% content SO, which was almost constant at 15 wt% SO. This nonlinear behavior of filled elastomers is known as the Payne effect [37]. This effect can be defined as the decrease of the storage or in-phase shear modulus with the increasing amplitude of oscillation [37]. The Payne effect is generated under a cyclic loading with small amplitude, indicating the dependence of the storage modulus on the amplitude strain [38]. The Payne effect increased with the increased stiffness of the materials, which correlated with the increasing concentration of filler materials in the composite [39]. Therefore, increasing stiffness in MRE should contribute to the occurrence of the Payne effect. As shown in Figure 6a, the Payne effect was obvious when performed during an on-state condition at 0 wt% content of SO. However, the effect was more prominent at 15 wt% content of SO.

This behavior occurs because, in the presence of a magnetic field of 600 mT, the interaction between the particles are much stronger, resulting in increased stiffness of the MRE. For the case of 15 wt% content of SO, the particle interactions are much stronger, due to less restriction from the elastomer matrix because of the softening effect of the SO. Consequently, this increases the stiffness of the MRE, contributing to a larger Payne effect. The reason for the Payne effect phenomenon is the formation of a network formed by filler–filler interactions (increased stiffness) in the MRE material. However, at much larger deformations of the MRE, the stress-induced MRE has broken down the filler network due to unstable particle bonding, which results in a decreasing shear storage modulus which can be deduced for the anisotropic MRE with 15 wt% SO at 600 mT, as compared to anisotropic MRE without SO [40]. This observation agrees with previous studies [41]. As shown in Figure 6c,d the loss factor of the sample without SO increases gradually with the strain increase and the increment is obvious for 0 wt% content of SO compared to 15 wt%, especially at the 600 mT data point for both isotropic and anisotropic MRE. In the case of anisotropic MRE, the loss factor increased from 0.1 to 1.1 MPa, and 0.3 to 0.4 MPa for both samples (0 and 15 wt% SO) at 600 mT. Usually, when the strain amplitude is large, the matrix undergoes a larger deformation. As more energy is dissipated, the loss factor increases with the increase in strain. When the content of the SO is increased, the lubricity of the network and CIPs on the substrate surface increases. This phenomenon results in an increase in the loss factor.

#### 2.3.3. Frequency Sweep

**Figure 7:**
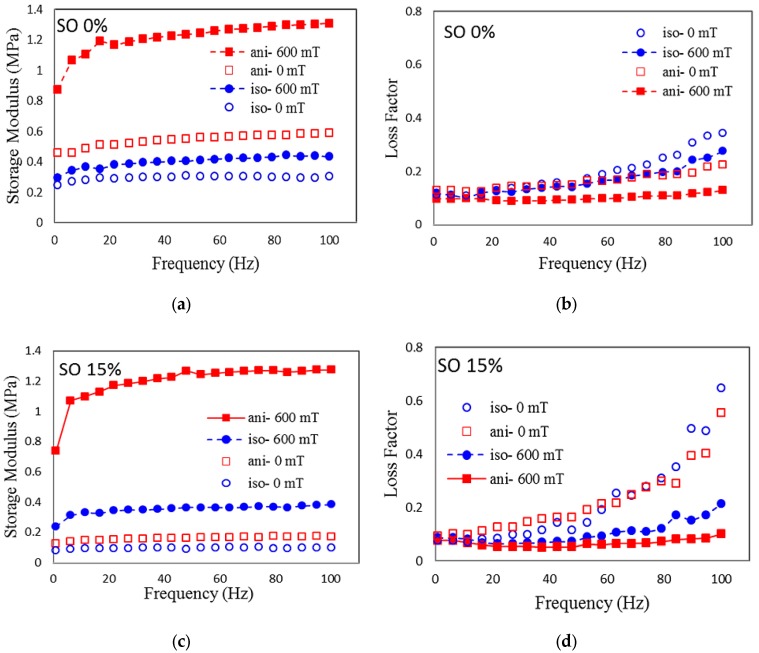
Storage modulus and loss factor of MRE with (**a**,**b**) 0 wt% of SO and (**c**,**d**) 15 wt% of SO as a function of excitation frequency at various magnetic fields.

MRE is commonly employed in vibration and acoustic devices; therefore, the effect of excitation of the frequency sweep is required to be conducted to evaluate the dynamic stiffness and damping properties of MRE. Therefore, in this work, selected plots of rheological properties are presented as a function of the oscillation frequency at different magnetic fields. Figure 7a–d demonstrates the effect of frequency at 0 and 600 mT of the magnetic field applied across the isotropic and anisotropic SO-based MREs for the lowest and highest SO content, 0 and 15 wt%, respectively. According to these figures, the same curve trend is observed, which shows the increment of all parameters dramatically in accordance with the increment of the frequency from 0.1 to 100 Hz. This tendency has been reported in several works [10]. In contrast to the storage modulus that is increased with the magnetic field, the loss factor shows a decreasing trend as the magnetic field intensity is increased. This occurrence happens because the MREs become stiffer as the frequency and the magnetic field are increased, while the damping ability of the samples is improved with the increment of these parameters.

In the case of isotropic MREs with 0 wt% SO, the shear storage modulus increased approximately from 0.25 to 0.30 MPa and 0.29 to 0.43 MPa at 0 mT and 600 mT, respectively. Meanwhile, for the anisotropic MRE with 0 wt% SO, the shear storage modulus increased from 0.18 to 0.58 MPa and 0.87 to 1.30 MPa at 0 mT and 600 mT, respectively. The increment of SO content up to 15 wt% contributes to a much higher shear storage modulus than 0 wt% SO, as depicted in Figure 7b. For the case of the isotropic sample, the increment of the shear storage modulus was 0.08 to 0.10 MPa at 0 mT and 0.24 to 0.38 MPa at 600 mT. On the other hand, for the anisotropic sample, the shear storage modulus increased from 0.13 to 0.17 at 0 mT and from 0.74 to 1.28 at 600 mT. The increment of the frequency and magnetic field from 0.1 to 100 Hz and 0 to 730 mT, respectively, resulted in an improvement of the inter-particle magnetic forces between CIPs. The effect was enhanced with the addition of SO due to the decrement of the initial shear storage modulus. It is noted that for anisotropic MREs, the initial shear storage modulus was higher due to the sample being much stiffer than the isotropic ones.

When the frequency was increased, the shear storage modulus could be increased since the deformation of the matrix molecular chain could not keep up with the change of the shear force. The dynamic response time of the samples decreased with increasing frequency. As a result, the stiffness of the whole system could be increased. At a particular frequency, for example, at 20 Hz, the shear storage modulus of MRE increased with an increasing magnetic field, which was due to the MR effect across all samples. On the other hand, the loss factor was enlarged as the frequency was increased up to 100 Hz. For the case of isotropic MREs, the loss modulus was increased from 0.12 to 0.28 MPa, and 0.09 to 0.21 MPa for both samples (0 and 15 wt% SO) at 600 mT. These results indicated that more energy was dissipated in the form of heat at higher frequencies, up to 100 Hz [42]. The dissipated energy was increased because the high friction occurred via interfacial slipping between CIPs, as well as between the CIPs and the silicone rubber matrix. The friction was then converted to kinetic energy, which was generated from the movement of shear into heat.

It was observed that more heat was also dissipated as the magnetic field increased at a much higher content of SO. This was due to the much softer matrix, which enhanced the friction between the CIPs and the matrix, resulting in a loss factor even higher for the off-state condition across both isotropic and anisotropic samples with 15 wt% SO [43]. Similar to the dissipated energy, the damping characteristic or the plasticity effect of the MREs showed the same trend and was matched by the research results obtained by Wu et al. [44]. Therefore, the incorporation of SO can contribute to the application of tunable damping properties of SO-based MREs for devices.

## 3. Sample Preparation

**Figure 8:**
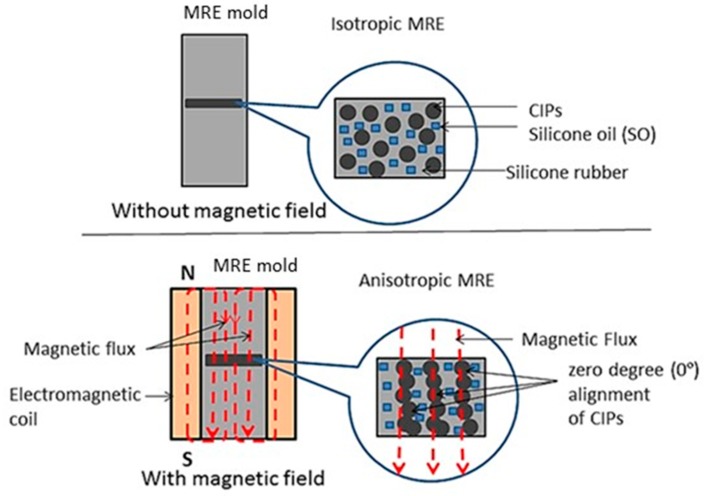
Illustration of the fabrication of the isotropic and anisotropic magnetorheological elastomers (MREs).

This study was conducted using silicone rubber, type RTV-two NS625/Nippon Steel from Tokyo, Japan, as the MRE matrix. It is a physically white liquid having a hardness of 22–50Å after complete curing. The magnetic particles used were carbonyl iron particles (CIPs) from BASF, Ludwigshafen, Germany, with an average size of 6 µm. The plasticizer used was silicone oil (SO), purchased from Nippon Steel, Tokyo, Japan. The densities of both the silicone rubber and CIP were 1.08 g/cm^3^ and 7.874 g/cm^3^, respectively, and the density of the SO was 1.26 g/cm^3^. Two groups of MRE samples were prepared with various concentrations of SO, namely, 5, 10 and 15 wt%. Each group was fabricated under two different curing conditions, isotropic and anisotropic, as shown in Figure 8. The chosen alignment angle for the anisotropic condition was 0-degree (lower part of Figure 8). The MREs that underwent both isotropic and anisotropic curing conditions were fabricated using the silicone rubber with 70 wt% CIP.

**Figure 9:**
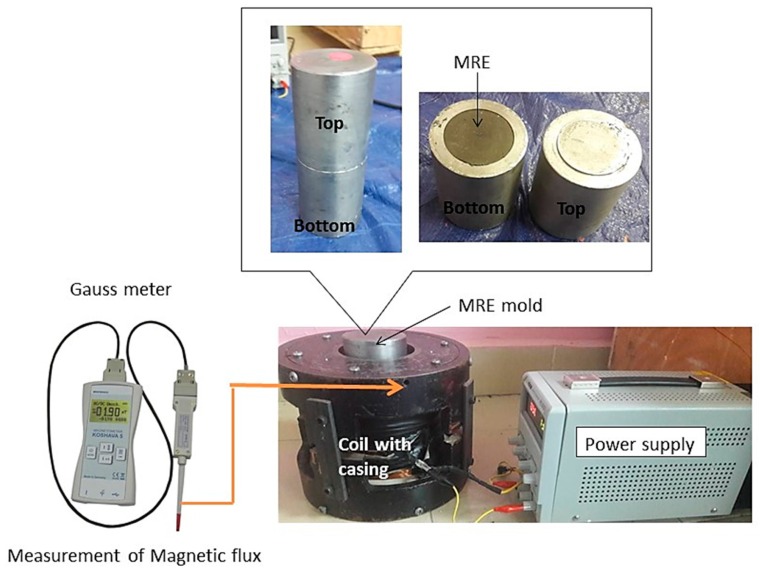
Anisotropic MRE using a closed-loop curing setup and diagram of flux density measurement.

The plasticizers were then added separately according to different concentrations of SO at 5, 10 and 15 wt%. The materials were mixed thoroughly and uniformly using a mechanical stirrer (WiseStir HT-DX, PMI-Labortechnik GmbH, Lindau, Switzerland) for 20 min. The mixtures were then added to the peroxide curing, and stirred again for one minute, for better homogenization. The curing agent was added during the curing process to transform the paste-like sample to that of a solid. Next, it was poured into a customized steel mold 1 mm thick and 70 mm in diameter. The mixture was allowed to solidify, under isotropic conditions. The solidification was done in the absence of magnetic fields. For the 0-degree anisotropic curing condition, the solidification was performed in the presence of a magnetic field, controlled by an applied current. The mold was placed at the center of the solenoid coil and a current of 0.5 A was applied, which was equivalent to 300 mT (refer to Figure 9). After 2 h of curing, the MRE sample was released from the mold and was ready to be tested and characterized.

## 4. Characterization

### 4.1. Tensile Test

A universal testing machine, AG-Xplus Series by Shimadzu, Kyoto, Japan, at the Malaysian Rubber Board, was used to conduct the tensile strength according to the standard ISO 37 test under uniaxial tension mode at a constant crosshead speed of 500 mm/min under room temperature. The standard dumbbell test pieces (75 mm (l) × 25 mm (gauge length) × 1.2 mm (w)) were die-stamped from tensile slabs with thicknesses of approximately 2.0 mm.

### 4.2. Morphological Test

The morphological characterization of the samples was observed under a scanning electron microscope (SEM, JEOL 7600F), Tokyo, Japan. The cross-section of the samples was observed at an acceleration voltage of 20 kV under magnifications up to 500 times. The images were then analyzed using Java image processing program (Image J, Version 1.52o, Oracle Corporation, Redwood Shores, CA, USA) analysis to determine the average distance between the particles inside the MREs.

### 4.3. Rheological Test

In the rheology test, all samples were examined in uncured and cured conditions which were related to viscosity and viscoelastic investigation, respectively. The viscosities of the uncured MREs with different mass ratios of SO were measured by using a rotational rheometer (Physica MCR 302 from Anton Paar, Graz, Austria). The samples were tested at 25 °C at a shear rate of 10^−1^ to 10 s^−1^, and the gap between the rotating disk was fixed at 1 mm. The dynamic viscoelastic properties of the MRE samples (cured MRE) were evaluated using the oscillatory rheometer MCR 302. All tests were conducted at room temperature. The rheometer MCR302 was equipped with the current controller MRD70 to generate the required magnetic field through the system, especially around the test area. The Viscotherm VT2 (Anton Paar, Graz, Austria) was utilized to control the temperature of the system at 25 °C.

The measurements were performed by sandwiching the MRE samples between a rotary disk and a parallel base plate, PP20 (Anton Paar, Graz, Austria). The dimensions of the samples were 20 mm in diameter and 1 mm thick. The tests were conducted in an oscillatory mode or under cyclic dynamic loadings. During this test, the viscoelastic materials deformed and returned to their original form after one cycle. The samples were tested under different flux densities from 0 to 800 mT. Both strain amplitude sweep and frequency sweep modes were conducted. For the strain amplitude sweep test, the frequency was 1 Hz and the strain amplitude swept from 0.001% to 10%. For the frequency sweep test, the shear strain amplitude was 0.01% and the frequency was swept from 0.1 to 100 Hz.

## 5. Conclusions

This work focused on the influence of SO, which affected the properties of MRE samples that were fabricated using a closed-loop mold enhancing the particle alignment to provide much better rheological properties. It was shown that the addition of SO in silicone rubber-based MREs could cause the decrement of the viscosity of the rubber matrix based on the uncured compound’s viscosity. The softer matrix helped CIPs to align well during the curing process, resulting in an anisotropic MRE. The tensile strength was reduced from 2.9 to 0.9 MPa (67%), as the SO content was increased from 0 to 15 wt%. The effect of additional SO toward the magneto-induced rheological properties was also investigated. The results revealed that MREs with the plasticizer additive exhibited a lower zero-field modulus, due to the changes in the rubber properties. The composition ratio of 70 wt% of CIPs, 15 wt% of silicone rubber, and 15 wt% of SO (70:15:15) brought about the increment of MR effects by 343% (0.3 MPa) for isotropic MREs and 646 % (0.8 MPa) for anisotropic MREs compared to the non-SO samples. The analysis implies that the matrix produced in this work exhibits the absolute modulus of 0.8 MPa, whereas the best reported so far has a value of 0.3 MPa. As expected, both the storage modulus and the loss factor increased as the excitation frequency and strain were increased. Therefore, we can conclude that the softening effect of SO helps the process improvement through the much simpler CIP incorporation and dispersion for the isotropic MRE, as well as the easier alignment of the CIPs for the anisotropic MRE. As a final note, some advantages of the proposed MREs will be validated across several device applications in the near future.

## Figures and Tables

**Table 1:** The zero-field storage modulus (*G*_0_), the magnetic induced modulus (∆*G*), and the MR effect of the MRE with different contents of SO.

No.	Sample	*G*_0_ (MPa)	*G_max_* (MPa)	Δ*G* (MPa)	Relative MR Effect (%)
1.	iso SO 0%	0.22	0.35	0.12	55
2.	iso SO 5%	0.13	0.22	0.09	65
3.	iso SO 10%	0.10	0.24	0.14	146
4.	iso SO 15%	0.08	0.37	0.29	343
5.	ani SO 0%	0.40	0.98	0.58	145
6.	ani SO 5%	0.20	0.85	0.65	330
7.	ani SO 10%	0.16	0.89	0.73	444
8.	ani SO 15%	0.12	0.92	0.79	646
